# Geographical differentiation of the *Euchiloglanis* fish complex (Teleostei: Siluriformes) in the Hengduan Mountain Region, China: Phylogeographic evidence of altered drainage patterns

**DOI:** 10.1002/ece3.2715

**Published:** 2017-01-13

**Authors:** Yanping Li, Arne Ludwig, Zuogang Peng

**Affiliations:** ^1^ The Key Laboratory of Freshwater Fish Reproduction and Development (Ministry of Education)Southwest University School of Life SciencesChongqingChina; ^2^ Department of Evolutionary GeneticsInstitute for Zoo and Wildlife ResearchBerlinGermany

**Keywords:** *Euchiloglanis*, genetic structure, Hengduan Mountain Region, phylogeny, phylogeography, Pleistocene glacial oscillations

## Abstract

The uplift of the Tibetan Plateau caused significant ecogeographical changes that had a major impact on the exchange and isolation of regional fauna and flora. Furthermore, Pleistocene glacial oscillations were linked to temporal large‐scale landmass and drainage system reconfigurations near the Hengduan Mountain Region and might have facilitated speciation and promoted biodiversity in southwestern China. However, strong biotic evidence supporting this role is lacking. Here, we use the *Euchiloglanis* fish species complex as a model to demonstrate the compound effects of the Tibetan Plateau uplift and Pleistocene glacial oscillations on species formation in this region. The genetic structure and geographical differentiation of the *Euchiloglanis* complex in four river systems within the Hengduan Mountain Region were deduced using the cytochrome *b* (*cyt b*) gene and 10 microsatellite loci from 360 to 192 individuals, respectively. The results indicated that the populations were divided into four independently evolving lineages, in which the populations from the Qingyi River and Jinsha River formed two sub‐lineages. Phylogenetic relationships were structured by geographical isolation, especially near drainage systems. Divergence time estimation analyses showed that the *Euchiloglanis* complex diverged from its sister clade *Pareuchiloglanis sinensis* at around 1.3 Million years ago (Ma). Within the *Euchiloglanis* complex, the divergence time between the Dadu–Yalong and Jinsha–Qingyi River populations occurred at 1.0 Ma. This divergence time was in concordance with recent geological events, including the Kun‐Huang Movement (1.2–0.6 Ma) and the lag time (<2.0 Ma) of river incision in the Hengduan Mountain Region. Population expansion signals were detected from mismatched distribution analyses, and the expansion times were concurrent with Pleistocene glacier fluctuations. Therefore, current phylogeographic patterns of the *Euchiloglanis* fish complex in the Hengduan Mountain Region were influenced by the uplift event of the Tibetan Plateau and were subsequently altered by paleo‐river transitions during the late Pleistocene glacial oscillations.

## 1 Introduction

Climatic fluctuations and geological events in the Pleistocene induced an accelerated change in the genetic structure of species and populations (Yan et al., 2013; Yu, Chen, Tang, Li, & Liu, 2014). The advancement and retreat of ice sheets in the Pleistocene led to population divergence and the generation of new lineages, as well as shaping population demographics. Specifically, the activity (or distribution) range of organisms was limited to refuges during the glacial periods, and later dispersed to open habitats during the interglacial periods. This repeated process shaped the geographical distribution of populations and the genetic variation within species, which, in turn, stimulated adaptation and allopatric speciation (Hewitt, 2000, 2004). Several studies have confirmed that species primarily distributed in the Tibetan Plateau region experienced population expansion after glacial retreat, suggesting that the eastern Tibetan Plateau might have been a refuge during the major Pleistocene glaciations (Qu & Lei, 2009; Qu, Lei, Zhang, & Lu, 2010). Gongga Mountain is located in the eastern region of the Tibetan Plateau, and it is a significant monsoonal maritime glacier center in the Hengduan Mountain Region. Quaternary glaciers remain widespread today, and glacial accumulation landforms are well preserved, due to the repeated glaciation of this region (Thomas, 1997). Glaciation cycles could drive the postglacial expansion of populations, thus shaping patterns in genetic variation (Li et al., 2009). However, geographical events have also markedly affected genetic differentiation in this region (Yu et al., 2014). The uplift of mountain systems and the formation of river systems could lead to isolating events that result in limited gene flow between populations, which would consequently provide opportunities for genetic diversification and speciation due to genetic drift and natural selection (Che et al., 2010; Streelman & Danley, 2003).

Freshwater fishes that are strictly constrained by drainage systems could provide unique insights into the relationships between current species distributions and the historical evolution of the paleo‐environment (Hewitt, 2004; Qi, Guo, Zhao, Yang, & Tangi, 2007). Historic basin connection events, which resulted from geological alterations, might have shaped the genetic structure of the fish population in the Hengduan Mountain Region (Durand, Templeton, Guinand, Imsiridou, & Bouvet, 1999). The main drainage trajectories have changed remarkably since the late Pliocene because the geomorphology has changed extensively (Clark et al., 2004). Researchers have advocated that the paleo‐drainage configurations of the main continental East Asian rivers that drain the southeastern section of the Tibetan Plateau were noticeably different to current patterns (Clark et al., 2004; He & Chen, 2006; Qi et al., 2015). The rivers that currently drain the plateau margin were once attributed to a single paleo‐Red River, which flowed southward and discharged into the South China Sea (Clark et al., 2004). River capture and reversal events related to the uplift of the Tibetan Plateau led to the subsequent reorganization of this river system into the current‐day major river drainage systems. The evolution of distribution patterns of primary freshwater fishes responded to the complex paleo‐geographical structure in the Tibetan Plateau, as well as to processes leading to their isolation or interconnection during the uplift event (Hurwood & Hughes, 1998; Montoya‐Burgos, 2003; Zhang et al., 2015).

The Hengduan Mountain Region lies on the southeast edge of the Tibetan Plateau, and it is considered as an important biodiversity hotspot because of its unique geological history and complex topography (Myers, Mittermeier, Mittermeier, da Fonseca, & Kent, 2000). The geomorphic evolution of this region resulted in the differentiation or isolation of many plant and animal populations (Fan et al., 2012). The Tibetan Plateau uplift resulted in major ecogeographical changes and hydrographic fluctuations; thus, species in the Hengduan Mountain Region represent appropriate models for examining the contributions of climate and geography to contemporary genetic diversification.

The *Euchiloglanis* fish species complex is composed of two species (*E. kishinouyei* and *E. longibarbatus*) that have high morphological similarity. This complex is part of a group of demersal freshwater catfish (Siluriformes: Sisoridae) that is distributed in the upstream region of the Yangtze River basin. The genetic divergence and population structure of the fishes are easily influenced by geographical events because of their weak mobility. Thus, the *Euchiloglanis* species complex is an ideal subject for investigating how paleo‐drainage shifts affect speciation in connection with the historic uplift of the Tibetan Plateau. However, morphology‐based taxonomy or molecular‐based phylogeny techniques have previously failed to recognize these fishes correctly (Guo, Zhang, & He, 2004; Zhou, Li, & Thomson, 2011). Consequently, the lack of a robust phylogenetic relationship for these fishes hindered detailed research on biogeography (Guo, He, & Zhang, 2007; Yu & He, 2012), and, hence, our understanding of the evolution of *Euchiloglanis* species in Southwestern China. In the current study, we considered the *Euchiloglanis* species distributed in the Hengduan Mountain Region as a species complex, and we attempted to determine the phylogeographical patterns of *Euchiloglanis* within this region. Based on the hypothesized links between paleo‐drainage systems in southeastern Tibet (Clark et al., 2004), we speculated several important geographical separations that might have shaped the patterns of *Euchiloglanis* distribution in this region.

We analyzed the geographical differentiation of the *Euchiloglanis* complex in the Hengduan Mountain Region, using complete sequences of mitochondrial cytochrome *b* (*cyt b*) gene and 10 microsatellite markers. Our goals were to: (1) infer the genetic structure and geographical differentiation of populations belonging to the *Euchiloglanis* species complex throughout the Hengduan Mountain Region; and (2) verify a vicariant speciation hypothesis (i.e. whereby the geographical range is split into discontinuous parts by the formation of a physical or biotic barrier to gene flow or dispersal) based on geological evidence of massive scale paleo‐drainage shifts that are related to the uplift of the Tibetan Plateau.

## 2 Materials and Methods

### 2.1 Sample collection

**Figure 1:**
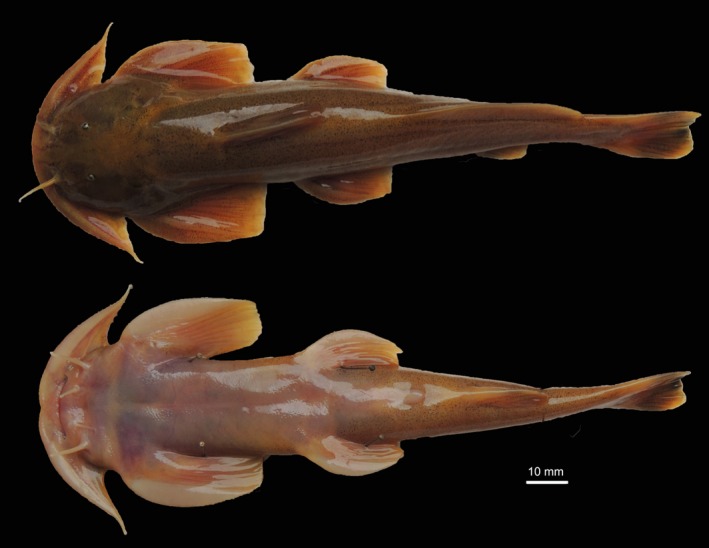
Dorsal and ventral view of the *Euchiloglanis*. The specimen was caught in the Dadu River, China

**Figure 2:**
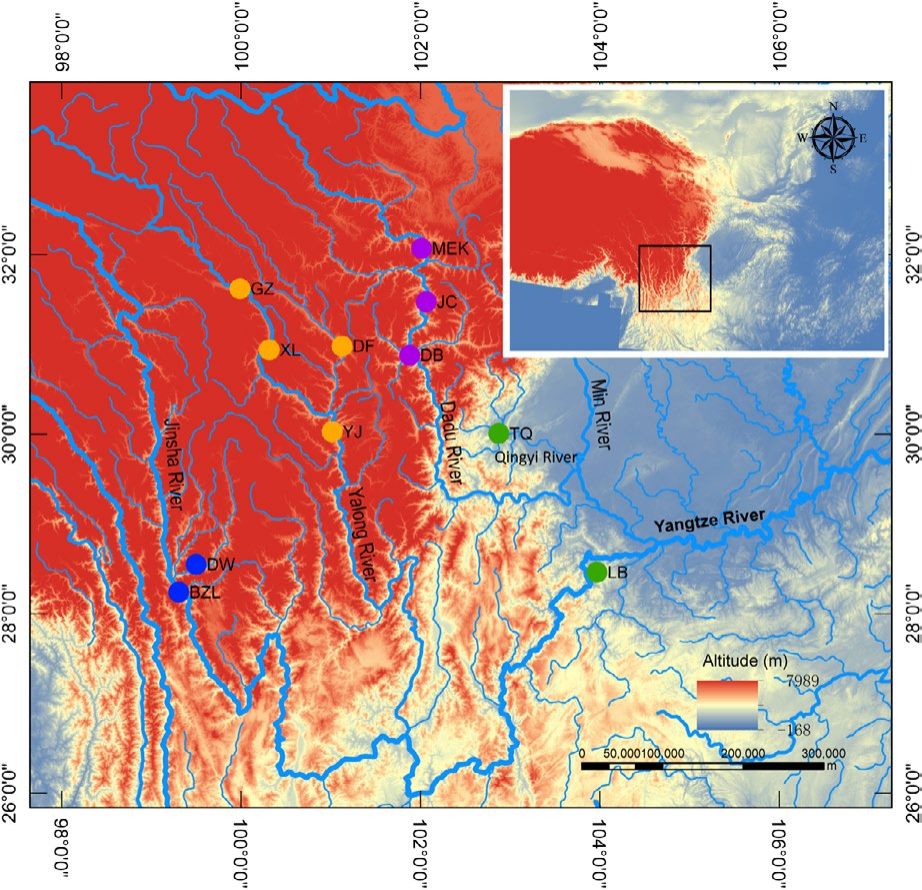
Sampling locations for *Euchiloglanis* in Hengduan Mountain Region. Different sites were colored according to the structure clusters. Location codes were consistent with those showed in Table 1

Samples were collected using fishhooks from 2012 to 2015. In all, 360 samples were gathered from 11 populations across four river systems, with 155 specimens being collected from the Dadu River, 117 from the Yalong River, 62 from the Jinsha River, and 26 from the Qingyi River (Table 1). A photograph of a *Euchiloglanis* fish specimen sampled from the Dadu River is shown in Figure 1. Detailed information about the sampling sites is presented in Figure 2. All individuals were used for mtDNA amplification, while 192 specimens from seven populations were selected for microsatellite genotyping. For microsatellite analyses, we chose specimens based on the range of the river. If the same river system contained more than two populations, we chose only two of them. Fin or muscle samples were preserved in 95% ethanol, and voucher samples were deposited in the Key Laboratory of Freshwater Fish Reproduction and Development (Ministry of Education), Southwest University School of Life Sciences, China. Sampling was performed according to the Chinese animal protection law.

**Table 1:** *Euchiloglanis* sampling localities, abbreviations, coordinates, sample size (N1 = *cyt b*, N2 = SSR), number of haplotypes (H), haplotype diversity (*H*_d_), nucleotide diversity (π), and Fu's *F*s and Tajima's D test of neutrality (*p < .05, **p < .001) based on mtDNA *cyt b* data

Sample sites	Abbreviations	Drainage	Coordinates	N1	N2	H	*H*_d_	π	Tajima's D	Fu's *F*s
Jinchuan, Sichuan	JC	Dadu River	31°28′37″N, 102°3′50″E	90	40	43	0.8861	0.0090	−1.423*	−13.406*
Danba, Sichuan	DB	Dadu River	30°52′44″N, 101°52′46″E	39	23	17	0.9163	0.0021	−1.461	−9.907**
Maerkang, Sichuan	MEK	Dadu River	31°99′45″N, 102°03′96″E	26		14	0.8800	0.0036	−1.075	−4.361*
Xinglong, Sichuan	XL	Yalong River	30°56′45″N, 101°18′62″E	10	33	8	0.9556	0.0039	0.564	−2.362
Yajiang, Sichuan	YJ	Yalong River	30°1'55′′N, 101°0′45′′E	41		11	0.7390	0.0026	−1.752*	−1.529
Daofu, Sichuan	DF	Yalong River	30°56′42′′N, 101°6′45′′E	31		11	0.6624	0.0013	−2.164*	−6.073**
Ganzi, Sichuan	GZ	Yalong River	31°37′4′′N, 99°59′12′′E	35	31	5	0.3580	0.0004	−0.924	−2.769*
Benzilan, Yunnan	BZL	Jinsha River	28°14′19′′N, 99°18′18′′E	45	30	6	0.6323	0.0007	−0.271	−1.804
Dongwang, Yunnan	DW	Jinsha River	28°32′56′′N, 99°39′52′′E	9		3	0.5556	0.0006	−0.064	−0.239
Leibo, Sichuan	LB	Jinsha River	28°26′99′′N, 103°57′59′′E	8	9	7	0.9643	0.0025	−1.283	−3.393*
Tianquan, Sichuan	TQ	Qingyi River	30°0′28′′N, 102°52′26′′E	26	26	9	0.8123	0.0012	−1.063	−4.127*

### 2.2 Laboratory protocols

Genomic DNA was extracted from fin tissues using the Qiagen DNeasy Kit (Qiagen, Shanghai, China), according to the instructions of the manufacturer. The *cyt b* sequences were amplified using previously described primers L14724 and H15915 (Xiao, Zhang, & Liu, 2001). PCR reactions were conducted in 25 μl volumes containing the following: 2.5 μl 10× buffer (Mg^2+^ free), 1.5 μl 50 mM MgCl_2_, 2.0 μl 2.5 mM dNTP, 1 U Taq DNA polymerase (rTaq, TaKaRa; Dalian, China), 1 μl 10 μM of each primer, 2–4 μl genomic DNA (50 ng/μl), and double‐distilled water added to make a final volume of 25 μl. The following conditions were used for PCR reactions: (1) pre‐denaturation at 95°C for 3 min; (2) denaturation at 95°C for 30 s; (3) annealing at 54–56°C for 30 s; (4) elongation at 72°C for 1 min (repeated 2–4 stages 35 times), and a final elongation at 72°C for 10 min. Negative controls (i.e., containing no DNA templates) were used in each PCR run, to test for contamination and artifacts. Reactions were performed in a Veriti Thermal Cycler (Applied Biosystems; Carlsbad, CA, USA). PCR products were tested via electrophoresis through 1% agarose gels and were purified with the Qiagen Gel Extraction Kit (Qiagen). Both strands of each product were sequenced using PCR primers.

Ten microsatellite loci (EK7, EK11, EK13, EK17, EK26, EK34, EK35, EK41, EK48, and EK66) were specifically developed for *E. kishinouyei* and were selected for genotyping analyses (Li, Wang, Zhao, Xie, & Peng, 2014). All loci were fluorescently labeled with FAM dye and amplified, as previously described (Li et al., 2014). An ABI 3730xl DNA Analyzer with ROX 500 was used as the internal size standard to determine the size of the PCR products. GENEMAPPER version 4.0 software (Applied Biosystems, USA) was used to score the allele designation.

### 2.3 Raw data processing

Forward and reverse directions of *cyt b* sequences were manually assembled using CONTIGEXPRESS version 3.0.0 (Invitrogen; Carlsbad, CA, USA). A multiple sequence alignment was performed with MAFFT version 6 (Katoh & Toh, 2008). SEAVIEW version 4 (Gouy, Guindon, & Gascuel, 2010) was used to edit the DNA sequences. The extent of variation in *cyt b* was determined by comparisons with sequences from other *Euchiloglanis* species. Haplotypes were defined with DNASP version 5.1 (Librado & Rozas, 2009). For microsatellite data, CONVERT version 1.3 (Glaubitz, 2004) was used to transform the input formats of the following programs: STRUCTURE, POPGENE, and ARLEQUIN. Before analysis, each locus was verified for deviation from the Hardy–Weinberg equilibrium, using POPGENE version 1.3.1 (Yeh & Boyle, 1997).

### 2.4 Genetic diversity and population differentiation

Genetic diversity indexes of *cyt b* and microsatellite loci were calculated. Regarding *cyt b*, genetic diversity parameters, including the number of polymorphic sites (s) and haplotypes (H), haplotype diversity (h), and nucleotide diversity (π), were calculated using DNASP. For microsatellite data, genetic diversity indices, including the number of alleles (*N*_A_), expected (*H*_E_) and observed (*H*_O_) heterozygosities, and the F‐statistics indices (*F*_IT_ and *F*_IS_), were assessed using POPGENE. Allelic richness (Rs) was computed using FSTAT version 2.9.3 (Goudet, 2001).

Genetic variation in the *Euchiloglanis* populations was also calculated. Pairwise population fixation indices for *F*_ST_ values among the 11 locations across the distribution range were performed using ARLEQUIN version 3.5 (Excoffier & Lischer, 2010) with 1,000 random permutations. The *F*_ST_ values of five groups were measured by comparing the genetic divergence at the drainage level. Population groups were defined according to phylogenetic analyses. In addition, the Mantel test measured in the R package “ade4” (Thioulouse, Chessel, Dole, & Olivier, 1997) was performed to compare the genetic distance [*F*_ST_/(1 − *F*_ST_)] to the geographical distance (ln·km) across populations for the *cyt b* gene and microsatellite loci. For each analysis, 100,000 randomizations were calculated. Moreover, analysis of molecular variance (AMOVA) was also computed in ARLEQUIN. Population groups were also divided according to phylogenetic analyses.

### 2.5 Phylogenetic analysis and population structure

The *cyt b* sequences of three *Pareuchiloglanis sinensis* individuals were amplified and used as outgroups because the species is the sister taxon of *Euchiloglanis*. *Glyptosternon maculatum* (DQ192471) was also chosen as an outgroup to avoid any bias. Before reconstructing the phylogenetic trees, an optimal DNA substitution model (GTR + I + G) was obtained based on model‐averaged parameters using the Akaike Information Criterion (AIC) in JMODELTEST version 2.1.4 (Darriba, Taboada, Doallo, & Posada, 2012).

Bayesian inference (BI), neighbor‐joining (NJ), and maximum parsimony (MP) were performed to reconstruct the phylogenetic tree among the *cyt b* haplotypes. Regarding BI, two independent Bayesian searches were conducted using MRBAYES version 3.2.1 (Ronquist et al., 2012), with one cold chain and three heated chains for the Markov chain Monte Carlo (MCMC) process, which began with random starting trees. The analysis was run for 1 × 10^6^ generations, and one tree per 100 generations was sampled for each run. The results of the BI analysis yielded 100,001 phylogenetic trees, with the first 25% representing burn‐in. Posterior probabilities were obtained from the 50% majority rule consensus tree of the remaining topologies. PAUP* version 4.0b10 was used to perform NJ and MP analyses (Swofford, 2002). Nodal support for NJ phylogram was calculated using 1,000 bootstrap replicates. For the MP analysis, a heuristic search strategy was employed with the tree bisection and reconnection branch‐swapping algorithm, including the random addition of taxa and 1,000 replicates per search. Nodal support for MP trees was evaluated using 1,000 bootstrap replicates. To visualize intraspecific genetic variation within *Euchiloglanis* better, the haplotype median‐joining network for *cyt b* was performed in NETWORK version 4.6.1 (Bandelt, Forster, & Rohl, 1999).

The genetic structure analyses of populations identified using the microsatellite loci were conducted using the Bayesian clustering analyses (Pritchard, Stephens, & Donnelly, 2000). Admixture models were chosen to assess possible clusters (*K* value). The lengths of the MCMC iterations were set to 50,000 with a burn‐in period of 5,000. The *K* value range was set to 1–7, and each *K* was replicated 20 times. The most likely *K* value was chosen according to peak value of the mean log likelihood [Ln *P*(*X*/*K*)] and the Delta *K* statistic for a given *K* (Evanno, Regnaut, & Goudet, 2005).

### 2.6 Divergence time estimation

Divergence times among the detected mitochondrial clades were evaluated in BEAST version 1.8.0 (Drummond, Suchard, Xie, & Rambaut, 2012), using an uncorrelated relaxed molecular clock Bayesian approach, following a lognormal distribution with the GTR + I + G substitution model proposed by JMODELTEST, in addition to a Yule prior approach and a random starting tree. The mean mutation rate was specified as a normal distribution, and estimates were calibrated using two age constraints. One constraint represented an upper bound of 4 Ma, derived from the capture of Tsangpo by the Brahmaputra River, which occurred before this time (Clark et al., 2004). The second internal time constraint was the divergence of *P. sinensis* and *E. davidi* (Peng, Ho, Zhang, & He, 2006). The internal time calibration was based on two branch points: (1) the divergence of *Pareuchiloglanis kamengensis* in the Yunnan population from *P. kamengensis* in the Tibetan population (1.3 ± 0.1 Ma) and (2) the divergence of *P. sinensis* from *E. davidi* (1.7 ± 0.3 Ma). The MCMC chain was run for 1 × 10^8^ generations and was sampled every 1,000 generations. The first 10% were burn‐in. TRACER version 1.5 (Rambaut & Drummond, 2007) was used to test the convergence of the chains to the stationary distribution, which was determined by an effective size (ESS) of more than 200 (Rambaut & Drummond, 2007). Moreover, three analyses with different random seeds were conducted to verify convergence. The corresponding tree files were merged with LOGCOMBINER1.8.0 (part of the BEAST package). TREEANNOTATOR version 1.8.0 was used to obtain a maximum credibility tree with the annotation of average node ages and the 95% highest posterior density (HPD) interval. Phylogenetic tree visualization was performed in FIGTREE version 1.4 (Rambaut & Drummond, 2012).

### 2.7 Historical demographic analyses

Demographic historical diversification in the population size of the *Euchiloglanis* complex in the Hengduan Mountain Region was explored using several approaches. Specifically, we completed neutrality tests, including Tajima's D (Tajima, 1989), Fu's *F*s tests (Fu, 1997), and R_2_ analyses (Ramos‐Onsins & Rozas, 2002). Pairwise differences between haplotypes and mismatch distributions were evaluated for each clade using ARLEQUIN. Sum of squares deviations (SSD) and raggedness statistics (Rag) significance values were evaluated with 10,000 permutations (Harpending, 1994). Mismatch distribution and neutrality tests, except R_2_, were calculated in ARLEQUIN, and R_2_ was performed in DNASP.

However, mismatch distribution and a neutrality test based on DNA data do not always catch historical signals because they depend only on the segregating sites and haplotype patterns (Fitzpatrick, Brasileiro, Haddad, & Zamudio, 2009). Therefore, the historical demographic dynamics of *Euchiloglanis* were deduced from Bayesian skyline plots (BSP) (Drummond et al., 2012), which were derived from three independent runs to recreate the demographic changes of five lineages identified based on the phylogenetic analyses. This recently developed coalescence‐based approach utilizes standard MCMC sampling procedures to evaluate the posterior probability distribution of ESS during intervals based on the HKY substitution model of sequence evolution for each individual clade (as determined by JMODELTEST). The model differed from that used in the phylogenetic analyses because model selection was run on each clade individually, and no outgroup taxa were included. The BSP of the five groups was evaluated using a strict molecular clock Bayesian approach, using BEAST with the Bayesian Skyline method and a random starting tree. Independent MCMC analyses were performed for 2 × 10^8^ generations with sampling every 2,000 generations, and 10% of the samples were burn‐in. To test for convergence, three analyses were performed for each clade with different random seeds. LOGCOMBINER was used to pool the replicate runs, with skyline plots being visualized in TRACER. ESS for all parameters was more than 200.

The results were consistent across runs, and a substitution rate of 2% was used in the *Euchiloglanis* c*yt b* region. Previously, the mtDNA substitution rate indicated that the speciation of a lacustrine fish species from its riverine ancestor (corrected based on mtDNA substitutions) was 0.02, which sufficiently pre‐dated the formation of the lake where speciation likely happened (Ovenden, White, & Adams, 1993; Waters et al., 2007).

## 3 Results

### 3.1 Genetic diversity and population differentiation

The 1,137‐bp c*yt b* sequences were obtained from each of the 360 individuals, and 125 haplotypes were recovered and deposited in the GenBank (Accession No. KX130459–KX130583). Overall, haplotype diversity (h = 0.9521 ± 0.0059) and nucleotide diversity (π = 0.01360 ± 0.00059) were relatively high. Among the 11 populations, the values of h and π of the GZ, BZL, and DW populations were lower than those observed in the remaining eight populations. h and π ranged from 0.3580 to 0.9643 and from 0.0006 to 0.0090, respectively (Table 1). For the microsatellite data, the number of alleles within all studied populations ranged from 4 (locus EK35) to 18 (locus EK7). The highest mean value of *H*_O_ was 0.602 (presented in the TQ population), and the lowest mean value was 0.178 (presented in the LB population). The mean values of *H*_O_ were lower than those of *H*_E_ in all populations, with the exception of the XL population (Table S1), suggesting a deficit in heterozygosity.

**Figure 3:**
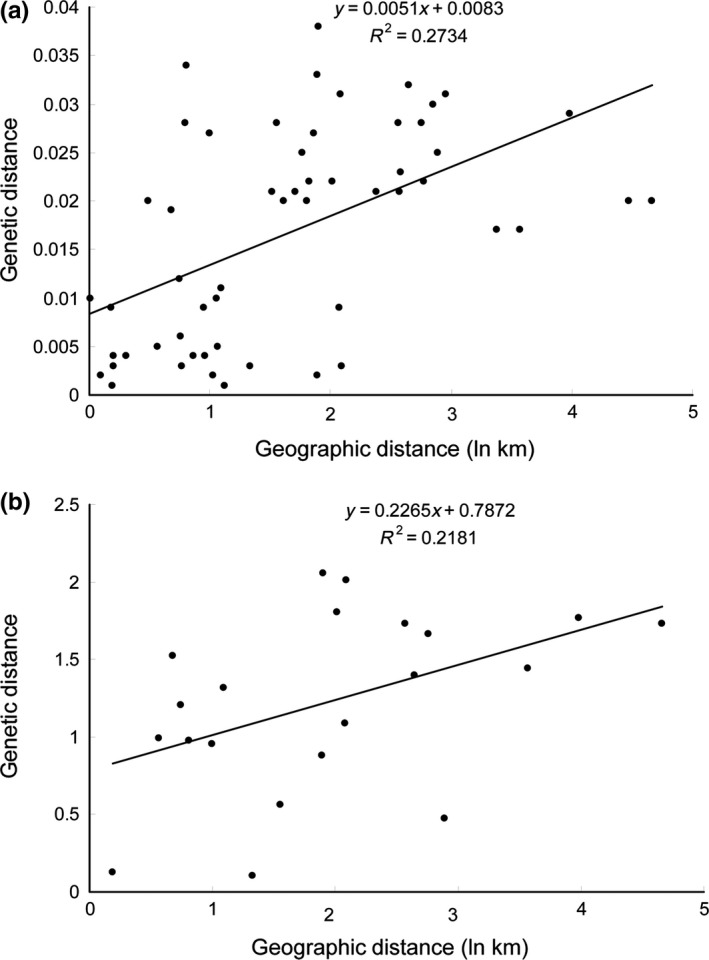
Scatter plots of genetic distance vs. geographical distance (km: kilometer) for pairwise population comparisons inferred from *cyt b* (a) and microsatellite data (b)

Among the 125 identified haplotypes, only seven (H3, H9, H23, H38, H48, H99, and H105) were shared by two or more populations from the same river. Moreover, the remaining haplotypes were restricted to one population, with 96 haplotypes being singletons (Table S2). These results indicate extensive genetic differentiation among populations. Pairwise *F*_ST_ analyses were conducted to further investigate the genetic differentiation among populations. For the mtDNA data, a significant difference was observed in all samples (*F*_ST_ = 0.80037, *p* < .001), indicating a high degree of geographical population divergence. Pairwise *F*_ST_ results suggested significant differentiation between any two populations (*p* < .001), except the BZL and DW populations and the GZ and DF populations (*p* > .05) (Table 2). The same pattern was observed for the microsatellite data, with significant divergence being found between any two populations (Table S3). In addition, the Mantel test generated *r* values of 0.523 (*p* = .0034) and 0.467 (*p* = .0461) for mitochondrial and microsatellite data, respectively, when evaluating the genetic diversity and geographical distance in the *Euchiloglanis* populations (Figure 3).

**Table 2:** Matrix of pairwise *F*_ST_ values of 11 populations inferred from mtDNA *cyt b* data

Population	JC	DB	MEK	BZL	DW	LB	DF	GZ	TQ	YJ	XL
JC											
DB	0.328**										
MEK	0.326**	0.204**									
BZL	0.784**	0.936**	0.920**								
DW	0.729**	0.915**	0.875**	−0.008							
LB	0.783**	0.930**	0.896**	0.949**	0.921**						
DF	0.366**	0.381**	0.381**	0.952**	0.943**	0.948**					
GZ	0.384**	0.456**	0.453**	0.970**	0.978**	0.974**	0.010				
TQ	0.790**	0.935**	0.913**	0.945**	0.935**	0.856**	0.950**	0.970**			
YJ	0.384**	0.336**	0.367**	0.922**	0.893**	0.915**	0.130**	0.176**	0.921**		
XL	0.359**	0.449**	0.381**	0.940**	0.894**	0.896**	0.326**	0.460**	0.928**	0.301**	

Significant pairwise differences: **p <* *.001. Populations are numbered as in Table 1.

### 3.2 Genetic structure

**Figure 4:**
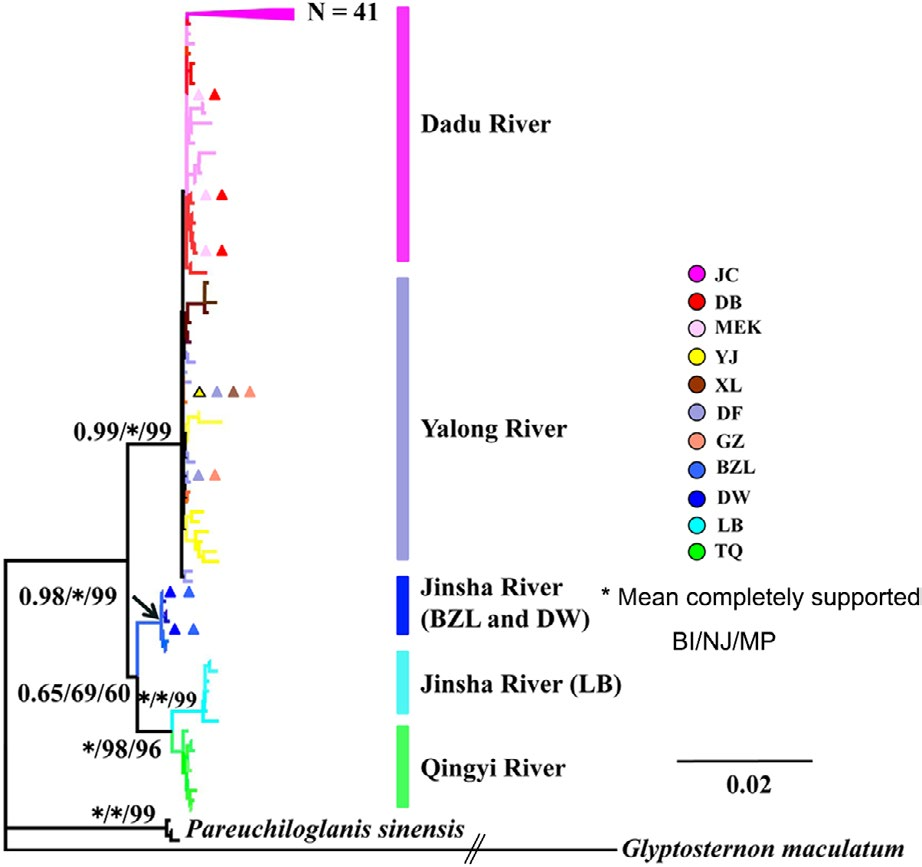
Phylogenetic relationships based on *cyt b* haplotypes. Numbers represented nodal supports inferred from Bayesian posterior probability (BI), neighbor‐joining probability (NJ), and maximum parsimony bootstrap analyses (MP), respectively. The supported or bootstrap value was only displayed among main clades. The symbol of “*” indicated a well‐supported Bayes posteriori possibility that reached a level of 1.0 or a significant bootstrap level of 100. *Glyptosternon maculatum* was used as an outgroup. Different colors do indicating different geographical locations

**Figure 5:**
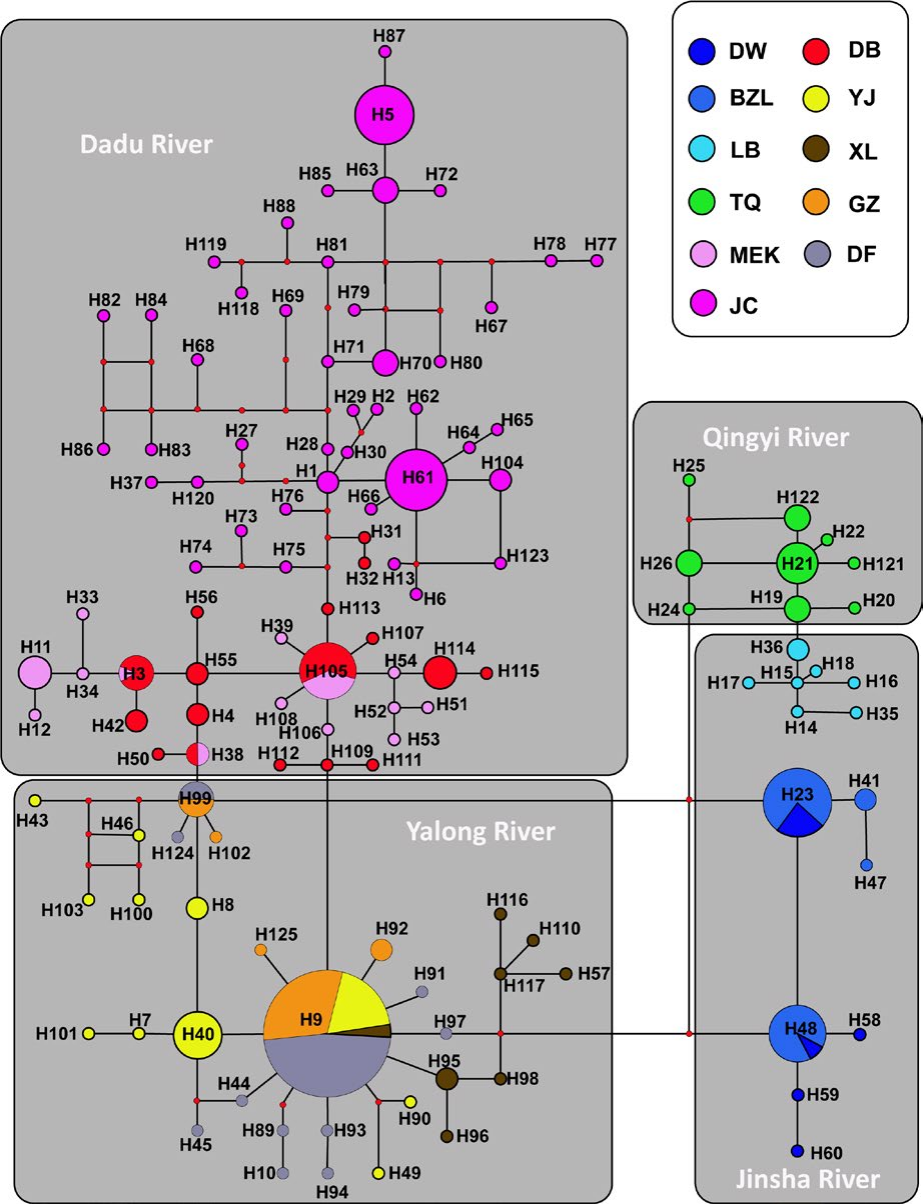
Median‐joining network of haplotypes identified in the *cyt b*. Haplotype numbers are consistent with those showed in Table S1. Circle sizes indicated the approximate numbers of individuals. Red dots represented number of nucleotide substitutions between haplotypes. Different colors indicated different geographical locations

The topologies of the BI, NJ, and MP trees were similar. Phylogenetic analyses based on haplotypes indicated that the *Euchiloglanis* complex was monophyletic (Figure 4). Phylogenetic trees constructed based on *cyt b* haplotypes and Bayesian genetic clustering analyses from microsatellite loci indicated that all populations were split into four independently evolving lineages, with the lineages appearing to reflect geographical associations linked to rivers. All haplotypes from the Dadu River (JC, DB, and MEK) formed one lineage, while all haplotypes from the Yalong River (YJ, XL, GZ, and DF) formed another lineage. Sichuan haplotypes from the Jinsha and Qingyi rivers were clustered into a single lineage with two sub‐lineages: (1) the haplotypes of the LB population in Sichuan Province, and (2) the haplotypes of the TQ population. The remaining lineage consisted of all Yunnan haplotypes from the Jinsha River (BZL and DW) (Figure 4). The haplotype networks were consistent with those deduced from the phylogenetic analyses (Figure 5).

**Figure 6:**
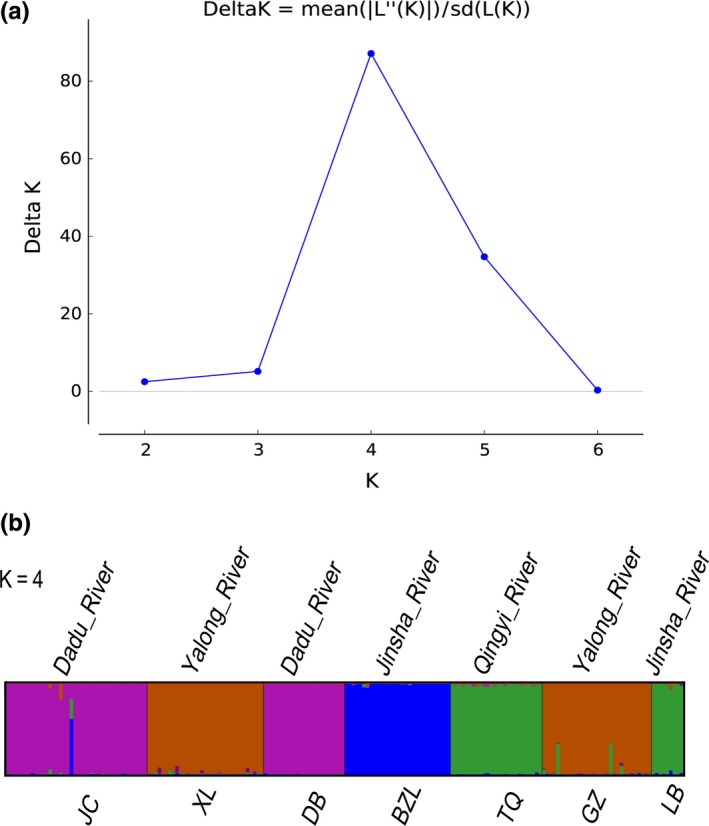
Structure clustering conducted based on microsatellite loci within populations of *Euchiloglanis*. (a) Delta *K* as a function of the *K* values according to 20 run outputs and (b) structure results at *K* = 4, with different colors indicating different clusters

Bayesian cluster analyses showed that the results of the structure analysis based on microsatellite loci were consistent with those of the phylogenetic analyses and haplotype networks based on mtDNA data. The whole population was split into four genetic clusters with a diverse maximum Δ*K* (Δ*K* = 87.13 at *K* = 4, Figure 6a). The relationships reflected the geographical associations with the rivers. The Qingyi River lineage was also closely related to the LB lineage from the Jinsha River (Figure 6b). Furthermore, hierarchical AMOVA indicated that differentiation among the lineages greatly contributed to the overall genetic variation observed in these populations. Specifically, hierarchical AMOVA explained 70.82% and 36.67% of total variation in the *cyt b* and microsatellite loci, respectively (Tables S4 and S5). The mean values of *F*_IS_ and *F*_IT_ were 0.0081 and 0.4632, respectively, based on microsatellite data (Table S6).

### 3.3 Estimation of divergence times

**Figure 7:**
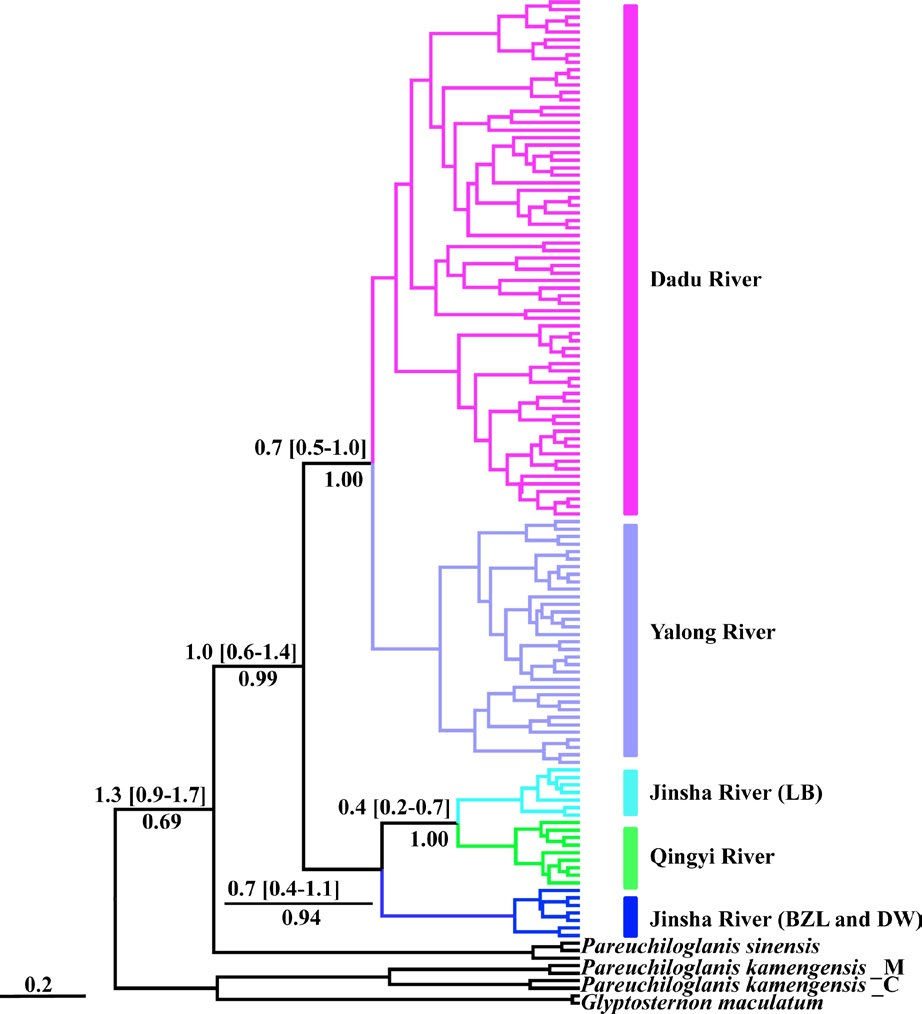
Divergence time estimation with time‐calibrated points was reconstructed from *cyt b* sequence. Digital numbers up branches indicated the time of species divergence events occurred (Ma: million years ago), following with the 95% credibility interval. Bayesian posterior probability was placed under divergence time labels

The divergence time analyses indicated that the ingroup diverged from *P. sinensis* at 1.3 Ma (95% HPD = 0.9–1.7). The split in the Dadu and Yalong Rivers was at 0.7 Ma (95% HPD = 0.5–1.0). The LB lineage from the Jinsha and Qingyi Rivers diverged at 0.4 Ma (95% HPD = 0.2–0.7). The divergence time of the LB lineage from the Jinsha–Qingyi Rivers and the BZL and DW lineages from the Jinsha River was also at 0.7 Ma (95% HPD = 0.4–1.1). Finally, the Dadu–Yalong lineage and Jinsha–Qingyi lineage diverged at 1.0 Ma (95% HPD = 0.6–1.4, Figure 7).

### 3.4 Historical demography

**Figure 8:**
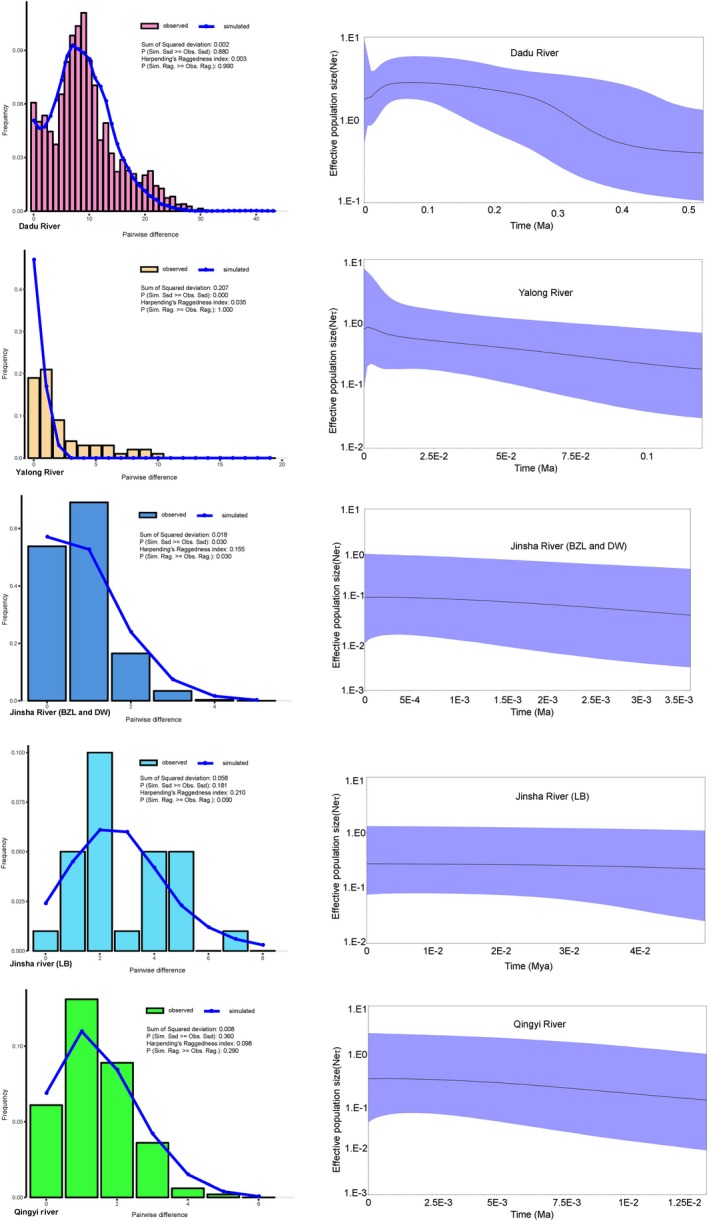
Mismatch distributions (left) and Bayesian skyline plots (right) of five population groups of *Euchiloglanis* inferred from mtDNA *cyt b* sequences. The Dadu River groups were analyzed according to sampled populations of JC, DB, and MEK. The Yalong River was computed based on the sampled populations of YJ, XL, DF, and GZ. The third group was calculated based on the sampled populations of BZL and DW. The other two groups were calculated based on the population of LB and TQ, respectively. For Bayesian skyline plots, the x‐axes were the time scale in million years, and the y‐axes were effective population size (units = Ne *τ, Ne represents the effective population size, τ represents generational time of the organism), the black line depicts the median population size, and the shaded areas represented the 95% confidence intervals of HPD analysis

Tajima's D and Fu's *F*s values associated with the Dadu River and Yalong River lineages were negative and highly significant (Table 3). The mismatch distributions for these two clades were unimodal (Figure 8). Moreover, the *p* values of Rag calculated for these two clades were above 0.05 (Figure 8). For the remaining groups, Tajima's D and Fu's *F*s values were negative, the R_2_ values were small and significant, and the mismatch distributions of these three groups were approximately unimodal, suggesting a weak signal of expansion in some parts of their ranges.

**Table 3:** Number of individuals (*N*), number of haplotypes (*H*), number of segregating sites (*S*), haplotype diversity (*H*_d_), haplotype diversity (π), Tajima's D and Fu's *F*s test of neutrality, Ramos‐Onsins and Rozas's R_2_ statistics (R_2_) (*p < .05, **p < .001), mismatch distribution, and the sum of squared deviations (SSD) and raggedness indexes (Rag) analyses for mtDNA *cyt b* sequences in five groups of *Euchiloglanis*

Groups	*N*	*H*	*S*	*H*_d_	π	Tajima's D	Fu's *F*s	R_2_	Mismatch distribution	SSD	Rag
Dadu River	155	71	120	0.9491	0.0082	−1.799*	−24.386**	0.084**	Unimodal	0.002	0.003
Yalong River	117	31	49	0.7218	0.0020	−2.364**	−24.741**	0.088**	Unimodal	0.207	0.035
Jinsha River (LB)	8	7	10	0.9643	0.0025	−1.2831	−3.393*	0.177**	Unimodal	0.058	0.210
Qingyi River	26	9	8	0.8123	0.0012	−1.063	−4.127*	0.122**	Unimodal	0.008	0.098
Jinsha River (BZL, DW)	54	7	5	0.6240	0.0007	−0.661	−2.716	0.105**	Unimodal	0.018	0.155
Total	360	125	196	0.9521	0.0136	−1.486*	−23.619*	0.074**	–	0.006	0.004

Bayesian skyline plots analyses showed a comparatively clear demographic history for the five divided clades (Figure 8), indicating that both the Dadu River and Yalong River underwent distinct population expansions, but recently experienced declining populations. The LB lineage from the Jinsha River was nearly stable after a prolonged period of slight population expansion. The Qingyi River exhibited a trend of population expansion over time. The BZL and DW from the Jinsha River revealed a tendency toward slightly increasing population size over time (Figure 8). The *x*‐axes of the BSP are in units of substitutions per site; therefore, the data could be transformed to determine the number of years before the present by dividing by the mutation rate. Thus, the BSP analyses indicate that the Dadu River and Yalong River experienced expansions at approximately 0.25–0.4 Ma and 0.005–0.3 Ma, respectively. The LB lineage and the BZL and DW lineages from the Jinsha River had slight expansions at 0.02–0.05 Ka (thousand years ago) and 0.5–3.5 Ka, respectively. The corresponding fluctuation time of the Qingyi River was 0.5–13 Ka.

## 4 Discussion

### 4.1 Phylogeographical structure

Phylogenetic analyses based on *cyt b* haplotypes indicated four independent evolutionary lineages, with one lineage being split into two sub‐lineages (Qingyi River and Jinsha River of LB). These results suggest that geographical isolation within the same drainage systems shaped the phylogenetic architecture of the populations. For instance, the Dadu River and Yalong River groups initially formed sister relationships and were subsequently clustered with the Jinsha River and Qingyi River groups. However, within the Jinsha group, samples from Yunnan (BZL and DW) did not cluster with the Jinsha River samples from Sichuan (LB). Moreover, the LB individuals from the Jinsha River had close relationships with individuals from the Qingyi River. This phenomenon might be caused by geographical isolation that allowed diverse populations to evolve in independent directions. The results of the Mantel tests based on both genetic markers suggested that the genetic distance was significantly correlated with the geographical distance of *Euchiloglanis*. Alternatively, the limited number of LB individuals might be the source of these differences. Thus, because of the topographic complexity and unique geological history of the Tibetan Plateau, it is essential to collect more specimens to reconstruct the relationship between geological events and the evolutionary history of endemic species. Moreover, the results of the phylogenetic analyses showed that individuals from the Dadu and Yalong rivers were not completely isolated. Thus, Dadu River group and Yalong River group might have originated from a single ancestral population, which subsequently separated because of crustal movements and river captures (Clark et al., 2004). In addition, the results showed high haplotype diversity (h = 0.9521 ± 0.0059) and nucleotide diversity (π = 0.01360 ± 0.00059) (h > 0.5 and π > 0.5%); therefore, the high differentiation between haplotypes might be ascribed to secondary contact between differentiated allopatric lineages and to the long evolutionary history of a large, steady population (Grant & Bowen, 1998). A haplotype network based on the *cyt b* data provided an enhanced visualization of intraspecific genetic variation within *Euchiloglanis* fishes. The results indicated that there were no shared haplotypes among tributaries or different reaches, suggesting that the Dadu and Yalong groups were completely isolated. Moreover, the Bayesian structure clustering analysis based on microsatellite data verified this pattern.

### 4.2 Divergence times and historic demography

Geological research has suggested that the evolutionary drainage systems of the Tibetan Plateau are marked by significant changes in paleo‐drainage patterns (Clark et al., 2004). The rivers that currently drain the plateau margin were historically a single paleo‐Red River that flowed southward and discharged into the South China Sea. However, the river patterns have drastically changed because of nearby river capture and drainage direction reversal (Barbour, 1936; Lee, 1933). In the middle Pliocene, the Jinsha River was insulated, with this isolation stimulating the genetic diversification of its inhabitants at the genus level. However, these populations subsequently expanded to the Yunnan and Sichuan rivers during the uplift event of the Himalayan region. Clark et al. (2004) suggested that the current Dadu River is most likely the product of an ancient river capture with the Anning River. The current Dadu River has a short, sharp segment that runs transversely to the main mountain range, and a relatively large, low‐gradient segment that flows parallel to or behind the main mountain range. Moreover, the high terraces of the Dadu River and low longitudinal river gradients on the Anning River potentially define a paleo‐longitudinal profile of the paleo‐Dadu/Anning River. Thus, the initially south flowing Anning River might have been captured by the high‐gradient river that became present‐day Dadu River (Barbour, 1936; Wang, 1998). The Dadu–Anning River capture point occurred near the anomalous place of high topography at and around Gongga Mountain (Clark et al., 2004). The Dadu and Anning transect differed by a middle depth of between 1500 and 2150 m under the relict landscape. Near the anomalous place of both transects, a fall in elevation of approximately 0.51 km in the current surface occurs locally across Xianshuihe (a tributary of the Yalong River) (Clark et al., 2005). Clark et al. (2005) concluded that the ages of the Danba and Yalong transect ranged from 10.5 to 8.4 Ma and 6.4 to 4.7 Ma, respectively. The origination ages of rapid river incision in Tibet were 13–9 Ma (Clark et al., 2005). Furthermore, a lag time of <2 Ma was calculated for the fluvial incision in the Hengduan Mountain Region (Tian, Kohn, Hu, & Gleadow, 2015). In the present study, we calculated the divergence times between the targeted populations from each river. When combined with the geological data, the results suggest that the river captures and reversals strongly influenced the current distribution of the *Euchiloglanis* complex in the Hengduan Mountain Region. Several molecular genetic analyses have tested the hypothesis of Quaternary divergence between fish populations resulting from vicariant isolation due to river capture (He & Chen, 2006; Qi et al., 2015), with the current study supporting this hypothesis.

Furthermore, the molecular clock results indicated that the divergence time was congruent with the Kun‐Huang Movement (1.2–0.6 Ma) and the extensive glacial period (EGP, 0.5–0.17 Ma). Zheng, Xu, and Shen (2002) stated that the Tibetan Plateau experienced four or five glaciation oscillations in the Quaternary (Zheng et al., 2002). Apart from the EGP that occurred at 0.5–0.17 Ma, the last glacial period (LGP) occurred at 0.08–0.01 Ma, and the last glacial maximum (LGM) occurred at 0.021–0.017 Ma (Shi, 1998). During the EGP, ice coverage permanently existed at high elevations and middle areas of the Tibetan Plateau (Shi, 2002; Yang, Rost, Lehmkuhl, Zhenda, & Dodson, 2004). The mismatch analyses and neutrality tests detected significant signals of rapid expansion, with R_2_ statistics being small and significant, suggesting recent demographic expansion in some parts of the range. Based on the results of the BSP analysis, the expansion time of the Dadu River and Yalong River groups were inferred to have occurred at approximately 0.25–0.4 Ma and 0.005–0.3 Ma, respectively. These times fall within the EGP. The expansion of the LB lineage of the Jinsha River of LB was inferred to occur at 0.02–0.05 Ma, which was congruent with the LGP (0.08–0.01 Ma). The Qingyi River underwent an expansion at 0.005–0.013 Ma, which was after the EGP (0.5–0.17 Ma), and possibly earlier than the LGM (0.021–0.017 Ma). Therefore, the results of this study provided evidence of the exceptional phylogeographical architecture of the *Euchiloglanis* in the Hengduan Mountain Region, which was initially shaped by the uplift event of the Tibetan Plateau. Furthermore, the results highlight the importance of paleo‐river connections, which were likely complicated by glacial movements in the late Pleistocene ice age. In conclusion, the divergence detected between the lineages in this study suggests that a number of speciation events might occurred in the Hengduan Mountain Region; however, to confirm these splits, large DNA datasets are required to resolve the phylogenetic relationships within the *Euchiloglanis* fish complex.

## Conflict of Interest

None declared.
