# Supplementary material for: Geographical differentiation of the Euchiloglanis fish complex (Teleostei: Siluriformes) in the Hengduan Mountain Region, China: Phylogeographic evidence of altered drainage patterns
Source: Ecol Evol. 2017 Jan 13;7(3):928–40. doi: 10.1002/ece3.2715 (PMC5288251; doi:10.1002/ece3.2715)
Supplement: Supplementary file 1 [file ECE3-7-928-s001.doc]

**Table S1.** Genetic diversity indices inferred based on the microsatellite data.

|  | EK7 | EK11 | EK13 | EK17 | EK26 | EK34 | EK35 | EK41 | EK48 | EK66 | Mean |
| --- | --- | --- | --- | --- | --- | --- | --- | --- | --- | --- | --- |
| JC |  |  |  |  |  |  |  |  |  |  |  |
| N_A_ | 6 | 8 | 5 | 4 | 5 | 8 | 4 | 11 | 6 | 3 | 6 |
| H_E_ | 0.628 | 0.729 | 0.565 | 0.304 | 0.446 | 0.582 | 0.501 | 0.817 | 0.367 | 0.439 | 0.538 |
| H_O_ | 0.550 | 0.625 | 0.675 | 0.325 | 0.400 | 0.400 | 0.525 | 0.825 | 0.350 | 0.450 | 0.513 |
| PIC | 0.554 | 0.672 | 0.483 | 0.273 | 0.387 | 0.519 | 0.405 | 0.783 | 0.340 | 0.359 | 0.477 |
| Rs | 4.005 | 4.782 | 3.245 | 2.578 | 2.984 | 4.124 | 2.626 | 6.619 | 3.326 | 2.4 | 3.669 |
| DB |  |  |  |  |  |  |  |  |  |  |  |
| N_A_ | 5 | 7 | 4 | 3 | 2 | 3 | 2 | 6 | 4 | 4 | 4 |
| H_E_ | 0.578 | 0.770 | 0.599 | 0.240 | 0.415 | 0.631 | 0.507 | 0.779 | 0.345 | 0.370 | 0.523 |
| H_O_ | 0.870 | 0.739 | 0.522 | 0.174 | 0.478 | 0.609 | 0.391 | 0.696 | 0.391 | 0.304 | 0.517 |
| PIC | 0.482 | 0.714 | 0.513 | 0.220 | 0.323 | 0.536 | 0.373 | 0.727 | 0.317 | 0.331 | 0.454 |
| Rs | 3.417 | 5.344 | 3.320 | 2.509 | 2.000 | 2.978 | 2.000 | 5.456 | 3.198 | 3.004 | 3.223 |
| XL |  |  |  |  |  |  |  |  |  |  |  |
| N_A_ | 8 | 5 | 3 | 2 | 4 | 2 | 3 | 4 | 7 | 3 | 4.1 |
| H_E_ | 0.753 | 0.633 | 0.592 | 0.373 | 0.558 | 0.451 | 0.243 | 0.548 | 0.556 | 0.416 | 0.512 |
| H_O_ | 0.788 | 0.485 | 0.576 | 0.424 | 0.758 | 0.485 | 0.273 | 0.667 | 0.485 | 0.455 | 0.539 |
| PIC | 0.714 | 0.582 | 0.503 | 0.300 | 0.453 | 0.346 | 0.217 | 0.448 | 0.509 | 0.367 | 0.444 |
| Rs | 5.763 | 4.126 | 2.934 | 1.997 | 2.895 | 2.000 | 2.207 | 2.895 | 4.239 | 2.794 | 3.185 |
| GZ |  |  |  |  |  |  |  |  |  |  |  |
| N_A_ | 9 | 6 | 3 | 3 | 3 | 2 | 2 | 3 | 5 | 4 | 4 |
| H_E_ | 0.759 | 0.640 | 0.539 | 0.535 | 0.535 | 0.337 | 0.032 | 0.515 | 0.354 | 0.534 | 0.460 |
| H_O_ | 0.677 | 0.613 | 0.258 | 0.355 | 0.645 | 0.290 | 0.032 | 0.581 | 0.323 | 0.355 | 0.413 |
| PIC | 0.717 | 0.595 | 0.459 | 0.418 | 0.418 | 0.277 | 0.031 | 0.428 | 0.315 | 0.314 | 0.397 |
| Rs | 6.087 | 4.749 | 2.885 | 2.500 | 2.500 | 1.994 | 1.290 | 2.756 | 2.848 | 2.767 | 3.038 |
| TQ |  |  |  |  |  |  |  |  |  |  |  |
| N_A_ | 7 | 7 | 6 | 5 | 3 | 3 | 3 | 4 | 3 | 6 | 4.7 |
| H_E_ | 0.738 | 0.833 | 0.704 | 0.794 | 0.271 | 0.432 | 0.619 | 0.563 | 0.639 | 0.644 | 0.624 |
| H_O_ | 0.731 | 0.846 | 0.615 | 0.769 | 0.308 | 0.211 | 0.692 | 0.539 | 0.615 | 0.692 | 0.602 |
| PIC | 0.680 | 0.793 | 0.652 | 0.744 | 0.239 | 0.381 | 0.524 | 0.453 | 0.548 | 0.576 | 0.559 |
| Rs | 5.315 | 6.327 | 4.958 | 4.900 | 2.306 | 2.920 | 2.960 | 2.923 | 2.986 | 4.203 | 3.980 |

**Table S1. Continue**

| LB |  |  |  |  |  |  |  |  |  |  |  |
| --- | --- | --- | --- | --- | --- | --- | --- | --- | --- | --- | --- |
| N_A_ | 4 | 4 | 4 | 3 | 2 | 3 | 1 | 3 | 5 | 4 | 3.3 |
| H_E_ | 0.686 | 0.660 | 0.706 | 0.392 | 0.209 | 0.392 | 0.000 | 0.569 | 0.791 | 0.477 | 0.488 |
| H_O_ | 0.556 | 0.444 | 0.222 | 0.000 | 0.000 | 0.222 | 0.000 | 0.111 | 0.111 | 0.111 | 0.178 |
| PIC | 0.593 | 0.579 | 0.607 | 0.340 | 0.178 | 0.340 | 0.000 | 0.468 | 0.709 | 0.421 | 0.424 |
| Rs | 4.000 | 4.000 | 4.000 | 3.000 | 2.000 | 3.000 | 1.000 | 3.000 | 5.000 | 4.000 | 3.3 |
| BZL |  |  |  |  |  |  |  |  |  |  |  |
| N_A_ | 5 | 4 | 3 | 3 | 4 | 3 | 2 | 5 | 2 | 5 | 3.6 |
| H_E_ | 0.248 | 0.394 | 0.292 | 0.506 | 0.610 | 0.463 | 0.481 | 0.617 | 0.066 | 0.688 | 0.436 |
| H_O_ | 0.233 | 0.400 | 0.000 | 0.000 | 0.333 | 0.533 | 0.500 | 0.633 | 0.000 | 0.600 | 0.323 |
| PIC | 0.236 | 0.358 | 0.260 | 0.445 | 0.556 | 0.366 | 0.361 | 0.548 | 0.062 | 0.624 | 0.382 |
| Rs | 2.992 | 3.098 | 2.467 | 2.946 | 3.846 | 2.300 | 2.000 | 3.579 | 1.514 | 4.062 | 2.880 |
| Total |  |  |  |  |  |  |  |  |  |  |  |
| N_A_ | 18 | 13 | 9 | 9 | 8 | 15 | 4 | 14 | 9 | 14 | 11.3 |
| H_E_ | 0.827 | 0.866 | 0.768 | 0.756 | 0.665 | 0.838 | 0.642 | 0.860 | 0.655 | 0.782 | 0.766 |
| H_O_ | 0.625 | 0.599 | 0.438 | 0.323 | 0.469 | 0.416 | 0.380 | 0.641 | 0.344 | 0.458 | 0.470 |
| PIC | 0.809 | 0.850 | 0.731 | 0.724 | 0.628 | 0.819 | 0.579 | 0.843 | 0.594 | 0.752 | 0.733 |
| Rs | 7.685 | 7.623 | 5.262 | 5.717 | 4.721 | 7.370 | 3.638 | 7.854 | 4.439 | 6.18 | 6.049 |

**Table S2.** Distribution of haplotypes among localities (Hap= Haplotype) based on mtDNA *cyt b* data **(**DDH=Dadu River, YLJ=Yalong River, JSJ= Jinsha River, QYJ= Qingyi River). Population codes are corresponding to Table 1.

| River | DDH | | | YLJ | | | | JSJ | | | QYJ |  |
| --- | --- | --- | --- | --- | --- | --- | --- | --- | --- | --- | --- | --- |
| Pop | JC | DB | MEK | YJ | XL | GZ | DF | BZL | DW | LB | TQ | Total |
| Hap 1 | 2 |  |  |  |  |  |  |  |  |  |  | 2 |
| Hap 2 | 1 |  |  |  |  |  |  |  |  |  |  | 1 |
| Hap 3 |  | 6 | 1 |  |  |  |  |  |  |  |  | 7 |
| Hap 4 |  | 4 |  |  |  |  |  |  |  |  |  | 4 |
| Hap 5 | 19 |  |  |  |  |  |  |  |  |  |  | 19 |
| Hap 6 | 1 |  |  |  |  |  |  |  |  |  |  | 1 |
| Hap 7 |  |  |  | 1 |  |  |  |  |  |  |  | 1 |
| Hap 8 |  |  |  | 4 |  |  |  |  |  |  |  | 4 |
| Hap 9 |  |  |  | 11 | 2 | 28 | 18 |  |  |  |  | 59 |
| Hap 10 |  |  |  |  |  |  | 1 |  |  |  |  | 1 |
| Hap 11 |  |  | 8 |  |  |  |  |  |  |  |  | 8 |
| Hap 12 |  |  | 1 |  |  |  |  |  |  |  |  | 1 |
| Hap 13 | 1 |  |  |  |  |  |  |  |  |  |  | 1 |
| Hap 14 |  |  |  |  |  |  |  |  |  | 1 |  | 1 |
| Hap 15 |  |  |  |  |  |  |  |  |  | 1 |  | 1 |
| Hap 16 |  |  |  |  |  |  |  |  |  | 1 |  | 1 |
| Hap 17 |  |  |  |  |  |  |  |  |  | 1 |  | 1 |
| Hap 18 |  |  |  |  |  |  |  |  |  | 1 |  | 1 |
| Hap 19 |  |  |  |  |  |  |  |  |  |  | 3 | 3 |
| Hap 20 |  |  |  |  |  |  |  |  |  |  | 1 | 1 |
| Hap 21 |  |  |  |  |  |  |  |  |  |  | 10 | 10 |
| Hap 22 |  |  |  |  |  |  |  |  |  |  | 1 | 1 |
| Hap 23 |  |  |  |  |  |  |  | 20 | 6 |  |  | 26 |
| Hap 24 |  |  |  |  |  |  |  |  |  |  | 1 | 1 |
| Hap 25 |  |  |  |  |  |  |  |  |  |  | 1 | 1 |
| Hap 26 |  |  |  |  |  |  |  |  |  |  | 5 | 5 |
| Hap 27 | 1 |  |  |  |  |  |  |  |  |  |  | 1 |
| Hap 28 | 1 |  |  |  |  |  |  |  |  |  |  | 1 |
| Hap 29 | 1 |  |  |  |  |  |  |  |  |  |  | 1 |
| Hap 30 | 1 |  |  |  |  |  |  |  |  |  |  | 1 |
| Hap 31 |  | 1 |  |  |  |  |  |  |  |  |  | 1 |
| Hap 32 |  | 1 |  |  |  |  |  |  |  |  |  | 1 |
| Hap 33 |  |  | 1 |  |  |  |  |  |  |  |  | 1 |
| Hap 34 |  |  | 1 |  |  |  |  |  |  |  |  | 1 |
| Hap 35 |  |  |  |  |  |  |  |  |  | 1 |  | 1 |
| Hap 36 |  |  |  |  |  |  |  |  |  | 2 |  | 2 |
| Hap 37 | 1 |  |  |  |  |  |  |  |  |  |  | 1 |
| Hap 38 |  | 2 | 2 |  |  |  |  |  |  |  |  | 4 |
| Hap 39 |  |  | 1 |  |  |  |  |  |  |  |  | 1 |
| Hap 40 |  |  |  | 18 |  |  |  |  |  |  |  | 18 |
| Hap 41 |  |  |  |  |  |  |  | 3 |  |  |  | 3 |
| Hap 42 |  | 2 |  |  |  |  |  |  |  |  |  | 2 |
| Hap 43 |  |  |  | 1 |  |  |  |  |  |  |  | 1 |
| Hap 44 |  |  |  |  |  |  | 1 |  |  |  |  | 1 |
| Hap 45 |  |  |  |  |  |  | 1 |  |  |  |  | 1 |
| Hap 46 |  |  |  |  |  | 1 |  |  |  |  |  | 1 |
| Hap 47 |  |  |  |  |  |  |  | 1 |  |  |  | 1 |
| Hap 48 |  |  |  |  |  |  |  | 19 | 2 |  |  | 21 |
| Hap 49 |  |  |  | 1 |  |  |  |  |  |  |  | 1 |
| Hap 50 |  | 1 |  |  |  |  |  |  |  |  |  | 1 |
| Hap 51 |  |  | 1 |  |  |  |  |  |  |  |  | 1 |
| Hap 52 |  |  | 1 |  |  |  |  |  |  |  |  | 1 |
| Hap 53 |  |  | 1 |  |  |  |  |  |  |  |  | 1 |
| Hap 54 |  |  | 1 |  |  |  |  |  |  |  |  | 1 |
| Hap 55 |  | 2 |  |  |  |  |  |  |  |  |  | 2 |
| Hap 56 |  | 1 |  |  |  |  |  |  |  |  |  | 1 |
| Hap 57 |  |  |  |  | 1 |  |  |  |  |  |  | 1 |
| Hap 58 |  |  |  |  |  |  |  |  | 1 |  |  | 1 |
| Hap 59 |  |  |  |  |  |  |  | 1 |  |  |  | 1 |
| Hap 60 |  |  |  |  |  |  |  | 1 |  |  |  | 1 |
| Hap 61 | 24 |  |  |  |  |  |  |  |  |  |  | 24 |
| Hap 62 | 1 |  |  |  |  |  |  |  |  |  |  | 1 |
| Hap 63 | 2 |  |  |  |  |  |  |  |  |  |  | 2 |
| Hap 64 | 1 |  |  |  |  |  |  |  |  |  |  | 1 |
| Hap 65 | 1 |  |  |  |  |  |  |  |  |  |  | 1 |
| Hap 66 | 1 |  |  |  |  |  |  |  |  |  |  | 1 |
| Hap 67 | 1 |  |  |  |  |  |  |  |  |  |  | 1 |
| Hap 68 | 1 |  |  |  |  |  |  |  |  |  |  | 1 |
| Hap 69 | 1 |  |  |  |  |  |  |  |  |  |  | 1 |
| Hap 70 | 2 |  |  |  |  |  |  |  |  |  |  | 2 |
| Hap 71 | 1 |  |  |  |  |  |  |  |  |  |  | 1 |
| Hap 72 | 1 |  |  |  |  |  |  |  |  |  |  | 1 |
| Hap 73 | 1 |  |  |  |  |  |  |  |  |  |  | 1 |
| Hap 74 | 1 |  |  |  |  |  |  |  |  |  |  | 1 |
| Hap 75 | 1 |  |  |  |  |  |  |  |  |  |  | 1 |
| Hap 76 | 1 |  |  |  |  |  |  |  |  |  |  | 1 |
| Hap 77 | 1 |  |  |  |  |  |  |  |  |  |  | 1 |
| Hap 78 | 1 |  |  |  |  |  |  |  |  |  |  | 1 |
| Hap 79 | 1 |  |  |  |  |  |  |  |  |  |  | 1 |
| Hap 80 | 1 |  |  |  |  |  |  |  |  |  |  | 1 |
| Hap 81 | 1 |  |  |  |  |  |  |  |  |  |  | 1 |
| Hap 82 | 1 |  |  |  |  |  |  |  |  |  |  | 1 |
| Hap 83 | 1 |  |  |  |  |  |  |  |  |  |  | 1 |
| Hap 84 | 1 |  |  |  |  |  |  |  |  |  |  | 1 |
| Hap 85 | 1 |  |  |  |  |  |  |  |  |  |  | 1 |
| Hap 86 | 1 |  |  |  |  |  |  |  |  |  |  | 1 |
| Hap 87 | 1 |  |  |  |  |  |  |  |  |  |  | 1 |
| Hap 88 | 1 |  |  |  |  |  |  |  |  |  |  | 1 |
| Hap 89 |  |  |  |  |  |  | 1 |  |  |  |  | 1 |
| Hap 90 |  |  |  | 1 |  |  |  |  |  |  |  | 1 |
| Hap 91 |  |  |  |  |  |  | 1 |  |  |  |  | 1 |
| Hap 92 |  |  |  |  |  | 1 |  |  |  |  |  | 1 |
| Hap 93 |  |  |  |  |  |  | 2 |  |  |  |  | 2 |
| Hap 94 |  |  |  |  |  |  | 1 |  |  |  |  | 2 |
| Hap 95 |  |  |  |  | 2 |  |  |  |  |  |  | 2 |
| Hap 96 |  |  |  |  | 1 |  |  |  |  |  |  | 1 |
| Hap 97 |  |  |  |  |  |  | 1 |  |  |  |  | 1 |
| Hap 98 |  |  |  |  | 1 |  |  |  |  |  |  | 1 |
| Hap 99 |  |  |  |  |  | 3 | 3 |  |  |  |  | 6 |
| Hap 100 |  |  |  | 1 |  |  |  |  |  |  |  | 1 |
| Hap 101 |  |  |  | 1 |  |  |  |  |  |  |  | 1 |
| Hap 102 |  |  |  |  |  | 1 |  |  |  |  |  | 1 |
| Hap 103 |  |  |  | 1 |  |  |  |  |  |  |  | 1 |
| Hap 104 | 4 |  |  |  |  |  |  |  |  |  |  | 4 |
| Hap 105 |  | 8 | 5 |  |  |  |  |  |  |  |  | 13 |
| Hap 106 |  |  | 1 |  |  |  |  |  |  |  |  | 1 |
| Hap 107 |  | 1 |  |  |  |  |  |  |  |  |  | 1 |
| Hap 108 |  |  | 1 |  |  |  |  |  |  |  |  | 1 |
| Hap 109 |  | 1 |  |  |  |  |  |  |  |  |  | 1 |
| Hap 110 |  |  |  |  | 1 |  |  |  |  |  |  | 1 |
| Hap 111 |  | 1 |  |  |  |  |  |  |  |  |  | 1 |
| Hap 112 |  | 1 |  |  |  |  |  |  |  |  |  | 1 |
| Hap 113 |  | 1 |  |  |  |  |  |  |  |  |  | 1 |
| Hap 114 |  | 5 |  |  |  |  |  |  |  |  |  | 5 |
| Hap 115 |  | 1 |  |  |  |  |  |  |  |  |  | 1 |
| Hap 116 |  |  |  |  | 1 |  |  |  |  |  |  | 1 |
| Hap 117 |  |  |  |  | 1 |  |  |  |  |  |  | 1 |
| Hap 118 | 1 |  |  |  |  |  |  |  |  |  |  | 1 |
| Hap 119 | 1 |  |  |  |  |  |  |  |  |  |  | 1 |
| Hap 120 | 1 |  |  |  |  |  |  |  |  |  |  | 1 |
| Hap 121 |  |  |  |  |  |  |  |  |  |  | 1 | 1 |
| Hap 122 |  |  |  |  |  |  |  |  |  |  | 3 | 3 |
| Hap 123 | 1 |  |  |  |  |  |  |  |  |  |  | 1 |
| Hap 124 |  |  |  |  |  |  | 1 |  |  |  |  | 1 |
| Hap 125 |  |  |  |  |  | 1 |  |  |  |  |  | 1 |

**Table S3.** Matrix of pairwise *F_ST_* values calculated from the microsatellite data among seven populations of *Euchiloglanis*. Population codes are corresponding to Table 1. Significant pairwise differences: **P < 0.001.

|  | JC | XL | DB | BZL | TQ | GZ | LB |
| --- | --- | --- | --- | --- | --- | --- | --- |
| JC |  |  |  |  |  |  |  |
| XL | 0.385** |  |  |  |  |  |  |
| DB | 0.081** | 0.367** |  |  |  |  |  |
| BZL | 0.456** | 0.480** | 0.474** |  |  |  |  |
| TQ | 0.301** | 0.230** | 0.299** | 0.406** |  |  |  |
| GZ | 0.396** | 0.081** | 0.377** | 0.490** | 0.233** |  |  |
| LB | 0.444** | 0.423** | 0.453** | 0.496** | 0.347** | 0.482** |  |

**Table S4.** Analyses of molecular variance (AMOVA) indicates degree of freedom (d.f.), Percentage variation (%), P value, and F*-*statistics among groups, among populations within groups and within populations, in various groups calculated on mtDNA *cyt b* data.

| Groups compared | No. groups | Source of variation | d.f. | Percentage variation (%) | P value | *F*-statistics |
| --- | --- | --- | --- | --- | --- | --- |
| All clades | 11 | Among groups | 4 | 70.82 | 0.000 | *F_SC_* =0.316 |
|  |  | Among populations within groups | 6 | 9.22 | 0.000 | *F_ST_* =0.800 |
|  |  | Within populations | 349 | 19.96 | 0.000 | *F_CT_* =0.708 |
| Dadu River | 3 | Among groups | 1 | 30.13 | 0.000 | *F_SC_* =0.081 |
|  |  | Among populations within groups | 1 | 5.68 | 0.000 | *F_ST_* =0.358 |
|  |  | Within populations | 152 | 64.19 | 0.320 | *F_CT_* =0.301 |
| Yalong River | 4 | Among groups | 2 | 24.89 | 0.179 | *F_SC_*= -0.011 |
|  |  | Among populations within groups | 1 | -0.83 | 0.131 | *F_ST_* =0.241 |
|  |  | Within populations | 113 | 75.94 | 0.000 | *F_CT_* =0.249 |
| Jinsha River and Qingyi River | 4 | Among groups | 2 | 93.71 | 0.385 | *F_SC_* =0.029 |
|  |  | Among populations within groups | 1 | -0.18 | 0.000 | *F_ST_* =0.935 |
|  |  | Within populations | 84 | 6.46 | 0.159 | *F_CT_* =0.937 |

**Table S5.** Analyze of molecular variance (AMOVA) for indicated degree of freedom (d.f.), sum of squares, variance components, percentage variation (%), and fixation indices among populations, among individuals within populations, and within individuals based on the microsatellite data.

| Source of variation | d.f. | Sum of squares | Variance components | Percentage of variation (%) | Fixation  Indices |
| --- | --- | --- | --- | --- | --- |
| Among  populations | 6 | 494.384 | 1.47824 Va | 36.67 | Fis=0.08395  (P<0.001) |
| Among loci  within populations | 185 | 511.929 | 0.21432 Vb | 5.32 | *F_ST_*=0.36671  (P<0.001) |
| Among samples within locus | 192 | 449 | 2.33854 Vc | 58.01 | Fit=0.41988  (P<0.001) |

**Table S6.** Summary of F-statistics and gene flow for *Euchiloglanis* based on ten microsatellite loci.

| Locus | Sample size | Fis | Fit | Fst | Nm* |
| --- | --- | --- | --- | --- | --- |
| EK7 | 384 | -0.0267 | 0.2519 | 0.2714 | 0.6712 |
| EK11 | 384 | 0.0882 | 0.3189 | 0.2529 | 0.7384 |
| EK13 | 384 | 0.2648 | 0.4765 | 0.2879 | 0.6184 |
| EK17 | 384 | 0.3341 | 0.6294 | 0.4434 | 0.3138 |
| EK26 | 384 | 0.0205 | 0.3994 | 0.3869 | 0.3962 |
| EK34 | 370 | 0.1445 | 0.5325 | 0.4535 | 0.3012 |
| EK35 | 384 | -0.0306 | 0.4905 | 0.5057 | 0.2444 |
| EK41 | 384 | 0.0604 | 0.3283 | 0.2851 | 0.6268 |
| EK48 | 384 | 0.2500 | 0.5428 | 0.3904 | 0.3904 |
| EK66 | 384 | 0.1042 | 0.4475 | 0.3833 | 0.4022 |
| Mean | 383 | 0.1181 | 0.4362 | 0.3607 | 0.4431 |

* Nm = Gene flow estimated from Fst = 0.25(1 - Fst)/Fst.
